# At-Line Reversed Phase Liquid Chromatography for In-Process Monitoring of Inclusion Body Solubilization

**DOI:** 10.3390/bioengineering8060078

**Published:** 2021-06-07

**Authors:** Julian Ebner, Diana Humer, Robert Klausser, Viktor Rubus, Reinhard Pell, Oliver Spadiut, Julian Kopp

**Affiliations:** 1 Research Division Integrated Bioprocess Development, Institute of Chemical, Environmental and Bioscience Engineering, Vienna University of Technology, 1060 Vienna, Austria; julian.ebner@tuwien.ac.at (J.E.); diana.humer@tuwien.ac.at (D.H.); robert.klausser@tuwien.ac.at (R.K.); viktor.rubus@tuwien.ac.at (V.R.); oliver.spadiut@tuwien.ac.at (O.S.); 2 SANDOZ GmbH, Mondseestrasse 11, 4866 Unterach, Austria; reinhard.pell@gmx.at

**Keywords:** inclusion bodies, inclusion body solubilization, tailored refolding, reversed phase liquid chromatography, process analytical technology tools, in-process monitoring, in-process control

## Abstract

Refolding is known as the bottleneck in inclusion body (IB) downstream processing in the pharmaceutical industry: high dilutions leading to large operating volumes, slow refolding kinetics and low refolding yields are only a few of the problems that impede industrial application. Solubilization prior to refolding is often carried out empirically and the effects of the solubilizate on the subsequent refolding step are rarely investigated. The results obtained in this study, however, indicate that the quality of the IB solubilizate has a severe effect on subsequent refolding. As the solubilizate contains chaotropic reagents in high molarities, it is commonly analyzed with sodium dodecyl sulfate polyacrylamide gel electrophoresis (SDS-PAGE). SDS-PAGE, however, suffers from a long analysis time, making at-line analytical implementation difficult. In this study, we established an at-line reversed phase liquid chromatography method to investigate the time-dependent quality of the solubilizate. To verify the necessity of at-line solubilization monitoring, we varied the essential solubilization conditions for horseradish peroxidase IBs. The solubilization time was found to have a major influence on subsequent refolding, underlining the high need for an at-line analysis of solubilization. Furthermore, we used the developed reversed phase liquid chromatography method for an in-process control (IPC). In conclusion, the presented reversed phase liquid chromatography method allows a proper control of IB solubilization applicable for tailored refolding.

## 1. Introduction

To date, approximately 20–30% of all approved biopharmaceuticals are produced in microbial hosts [1,2]. Insoluble aggregates, better known as inclusion bodies (IBs), produced by the gram-negative bacterium *Escherichia coli*, present a dominant fraction of the microbial production segment [3]. This is mainly because cultivation with *E. coli* can be carried out at very low costs in short fermentation run-times and high target protein concentrations at a high purity can be achieved [4,5,6]. Early downstream steps in IB processing, however, are notorious for their high complexity and low yields [7,8]. In particular, refolding is regarded as a major bottleneck in IB processing. Solubilization and refolding strategies are commonly developed empirically with protocols being highly dependent on the target protein [9,10]. Protein hydrophobicity, for instance, affects the required molarity of the chaotropic agent and pH in solubilization and refolding [11]. The addition of reducing agents during solubilization is required for proteins containing disulfide bonds [8], subsequently influencing the amount of oxidizing compounds to be added in refolding [12]. Refolding yields are highly dependent on the protein in question as well as the protein concentration during the refolding process: only 15–25% of refolding yields are reported for many therapeutic proteins [13] compared with a refolding yield of 97% for the enzyme lysozyme [14].

Singh et al. reported that mild solubilization boosts the refolding yield [15]. The reduction of chaotropic reagent molarity was compensated by either highly alkaline conditions or via the addition of solubilization-enhancing chemicals (i.e., n-propanol) [16,17]. The analyses in these studies, however, were performed with the commonly known sodium dodecyl sulfate polyacrylamide gel electrophoresis (SDS-PAGE) [17]. SDS-PAGE is frequently used for a solubilizate analysis as it tolerates the harsh conditions [18,19]. Various staining protocols (e.g., silver staining or Coomassie staining) with a different selectivity and treatment time have been established [20] and subsequent immunoblotting can further increase the sensitivity of gel-based analytical methods [21].

Unfortunately, this gel-based method is not suitable for in-process control (IPC) as the sample treatment, method running time and staining protocols are very time-consuming. However, according to quality by design (QbD) principles in pharmaceutical manufacturing, an IPC must be applied [22] and, for this purpose, process analytical technology (PAT) tools are of a great advantage as they allow a timely process intervention [23,24]. High molarities of chaotropic reagents required for solubilization, however, interfere with the measuring principles of many available PAT tools (i.e., near-infrared and Raman spectroscopy) [25,26].

Several liquid chromatography (LC) separation principles can tolerate the high molarities of the chaotropic agents required for the sample dissolution [27,28]. LC is implemented for the quantification of diverse biopharmaceuticals due to the straightforward and facile sample preparation as well as a rapid and accurate analysis [29,30,31]. Hydrophobic interaction chromatography (HIC), ion exchange chromatography (IEX) and size exclusion chromatography (SEC) are frequently used in a recombinant protein analysis [32,33]; however, high molarities of the chaotropic reagent required in IB solubilization again complicate the implementation of these chromatographic techniques. Reversed phase liquid chromatography (RPLC) is a denaturing chromatographic technique tolerating these harsh conditions [18]. Furthermore, RPLC is known for its robustness, high selectivity and compatibility with a mass spectrometric analysis [34,35] making it a highly suitable technique for in-process monitoring in IB processing.

In this study, we implemented an RPLC method applicable for an at-line solubilizate analysis. In this context, at-line defines a measurement that is performed in close proximity to the process stream as specified by regulatory agencies [36]. To demonstrate the need for in-process monitoring, we used HRP (horseradish peroxidase) as a model protein. HRP contains four disulfide bonds, thus requiring a complex solubilization and refolding strategy. We hypothesize that RPLC is faster and more precise than SDS-PAGE to determine the optimal solubilization conditions for tailored refolding. Hence, the solubilization conditions for HRP were varied and an SDS-PAGE analysis was compared with an RPLC analysis. The obtained results confirmed the effect of the solubilizate on the subsequent refolding step. In addition, the in-process control based on at-line RPLC measurements was demonstrated. The results of this study demonstrate that solubilizate quality influences the refolding yield and RPLC is suitable for the IPC of IB solubilization.

## 2. Materials and Methods

### 2.1. Production and Isolation of HRP IB

HRP was produced in *E. coli* BL21 (DE3) (Life Technologies, Carlsbad, CA, USA) with details stated in previous publications [37,38]. After a successful HRP expression [37,39,40], the cell broth was harvested via centrifugation and the biomass was stored at −20 °C.

Cell disruption was carried out at 1200 bar for 3 passages using a high-pressure homogenizer (PANDA+ 2000, GEA, Biberach, Germany). After centrifugation at 10,000 rpm, 4 °C and 20 min (Eppendorf, Hamburg, Germany), the soluble fraction was discarded. The resulting IB pellet was washed two times with a buffer (50 mM Tris, pH 8, 500 mM NaCl, 2 M Urea). The IB pellets were then resuspended in water, aliquoted to a defined wet IB weight, centrifuged (20,379 rcf, 4 °C, 20 min) and stored at −20 °C until further use.

### 2.2. Solubilization and Refolding

#### 2.2.1. In-Process Monitoring

The aliquoted IBs were thawed and resuspended in an HRP solubilization buffer (50 mM Tris/HCl pH 8; 6 M Urea). After the resuspension, DTT (1 M DTT stock) was added to a final concentration of 0 mM, 7.11 mM and 14.22 mM, respectively. The solubilization was performed at RT and a slight agitation and the samples were drawn after 0.5 h, 2 h, 4 h, 6 h, 8 h and 21 h. The samples were centrifuged (20,379 rcf, 4 °C, 20 min) and the supernatant was used for refolding, the RPLC analysis and diluted 1:2 in 2x Laemmli buffer for the SDS-PAGE analysis. For refolding, the solubilizate was diluted 1:40 in a pre-cooled HRP refolding buffer (20 mM Tris/HCl pH 8.5; 2 M Urea; 2 mM CaCl_2_; 7% *v/v* glycerol) containing either 0 mM, 1.27 mM or 2.54 mM GSSG (glutathione disulfide) [37]. Refolding was performed for 48 h at 4 °C on a rocker-shaker. Hemin was added 24 h after the refolding start to a final concentration of 20 µM (1 mM Hemin stock in 100 mM KOH). After refolding, the enzyme activity was measured as described previously [37].

#### 2.2.2. Demonstration of At-Line RPLC for IPC

In order to demonstrate the applicability of the at-line RPLC method for the IPC, we produced two different IB batches. Fermentations for both batches were conducted as described here [39] only the specific feeding rate (q_s_) during the induction was varied. Batch 1 was conducted at a q_s_ of 0.25 g/g/h during the induction whereas Batch 2 was performed at a q_s_ of 0.35 g/g/h during the induction. The harvest, cell disruption, IB wash, aliquoting and storage were done identically for both batches.

The inclusion bodies from both batches were solubilized at a concentration of 100 g wet IB/L solubilization mix. The solubilization buffer consisted of 50 mM glycine pH 10; 6 M Urea. DTT was added to a final concentration of 7.11 mM (1 M DTT stock). The solubilization was performed for 0.5 h at RT on a rocker-shaker. After centrifugation (20,379 rcf, 4 °C, 20 min), the supernatant was analyzed using the described RPLC method. Batch 1 solubilizate was diluted 1:40 in a refolding buffer (20 mM glycine pH 10; 2 M Urea; 2 mM CaCl_2_; 7% *v*/*v* glycerol; 1.27 mM GSSG). For IB Batch 2, two different refolding approaches were performed: the solubilizate was diluted 1:40 (= “fixed dilution”) and the dilution was adapted based on the RPLC results in order to achieve the same monomeric HRP concentration as in Batch 1. In this case, the dilution was reduced to 1:17 due to a lower concentration of the target protein HRP in IB Batch 2.

### 2.3. Analytical Techniques

#### 2.3.1. Reversed Phase Liquid Chromatography Measurements

The RPLC measurements were performed using a Dionex UltiMate 3000 system with a quaternary solvent delivery pump, an auto-sampler with a sample thermostat and a UV detector (Thermo Fisher, Waltham, MA, USA). The wavelength for the UV detection was set to 280 nm in order to monitor the protein absorption allowing the quantification of the target protein and its impurities. The instrument control and data acquisition were carried out via Chromeleon 7.2 software (Thermo Fisher). Prior to the RPLC measurement, all samples were centrifuged (20,379 rcf, 4 °C, 20 min) to separate the aggregates from the soluble fraction. We used a BioResolve RP mAb Polyphenyl column (dimensions 100 mm × 3 mm, particle size 2.7 µm) (Waters Corporation, MA, USA) connected to a pre-column (3.9 mm × 5 mm, 2.7 µm) of the same stationary phase. The mobile phase was composed of ultrapure water (MQ; eluent A) and acetonitrile (eluent B) both supplemented with 0.1% (*v*/*v*) trifluoroacetic acid. Ultrapure water was acquired from a Milli-Q system from Merck Millipore (Darmstadt, Germany). Acetonitrile (HPLC-grade) and trifluoroacetic acid (TFA, >99.9%) were obtained from Carl Roth (Karlsruhe, Germany). For the analysis, a recently published RPLC method, which had been developed and validated according to QbD principles, was modified in terms of gradient, column temperature and flow rate [18]. The method was optimized empirically, reducing the overall running time to 8.1 min in order to allow for short analysis times during solubilization. The runs were conducted at 78 °C and a flow rate of 1.2 mL/min using the gradient displayed in Table 1.

The HRP concentration in the solubilizate was determined using a bovine serum albumin (BSA) standard calibration ranging from 0.0625 g/L to 1.0000 g/L. The BSA was used for the quantification as no non-glycosylated standard was available for HRP.

#### 2.3.2. SDS-PAGE Measurements

For the SDS-PAGE analysis, the samples were mixed with 2× concentrated Laemmli buffer to achieve a 1× concentration of Laemmli buffer in the final dilution. The used buffer solution did not contain β-mercaptoethanol (non-reducing conditions) in order to analyze the solubilization quality in regard to disulfide bridge formation. The samples were heated to 95 °C for 10 min. A total of 5 µL of each sample was loaded onto pre-cast SDS gels (4–15%, Bio-Rad, Hercules, CA, USA). The gels were run in a Mini-PROTEAN Tetra System (Bio-Rad) for 30 min at 180 V and stained with Coomassie Blue. The protein bands were evaluated densitometrically using ImageLab software (Bio-Rad).

#### 2.3.3. HRP Enzymatic Activity Assay

The HRP enzyme activity was measured with a Tecan Infinite M200 PRO (Männedorf, Switzerland) using flat-bottom polystyrene 96-well plates, as described previously [37]. The samples after refolding were diluted in the range of 1:2–1:25 in a dilution buffer (20 mM Bis-Tris; pH 7; 7% *v*/*v* glycerol) depending on their volumetric activity. A total of 170 µL of ABTS solution (50 mM KH_2_PO_4_; pH 5; 5 mM ABTS) was mixed with 10 µL of the respective diluted sample in the well. The reaction was started by adding 20 µL hydrogen peroxide (the final concentration in the well was 1 mM). Immediately after the start of the reaction, the change in the absorbance at 420 nm was recorded at 30 °C for 2 min. The volumetric enzyme activity was calculated using Equation (1):

(1) $$A\left[\frac{U}{mL}\right]=\frac{V_{total}*∆\frac{A}{min}*Dilution}{V_{\mathrm{sample}}*d*\varepsilon }$$

*V_total_*—total well volume (μL).

∆*A*/*min*—change in absorption (∆_Abs_ 420 nm/min).

*Dilution*—dilution of the sample.

*V_sample_*—volume of the sample (µL).

d—length of the beam path through the well (d = 0.58 cm).

ε—extinction coefficient (ε_420_ = 36 mM^−1^ cm^−1^).

### 2.4. Experimental Design

The experiments were conducted using a full factorial design, as shown in Table 2, varying the solubilization time and DTT concentrations in a multivariate data approach. All combinations of the solubilization time and DTT concentration are listed in Supplementary Table S1. As responses for this design of experiment (DoE), the concentration of monomeric HRP was quantified with SDS-PAGE and RPLC. Additionally, all solubilization conditions were refolded at a GSSG concentration of either 0 mM, 1.27 mM or 2.54 mM GSSG to verify the effect on the refolding yield.

### 2.5. Multivariate Data Assessment of Solubilization and Refolding

An analysis of the used design of experiments (DoEs) was done using a multivariate data assessment program (MODDE 12, Umetrics, Sweden) with the model being based on a multiple linear regression. The results were analyzed for the statistical relevance of the model by the measure of fit (R^2^) and the model predictability (Q^2^).

## 3. Results and Discussion

### 3.1. In-Process Monitoring

In order to be applicable as an in-process monitoring method for solubilization, the respective analytical method had to meet three criteria:

1. The ability to quantify a defined quality attribute;
2. The quality attribute had to influence the refolding behavior;
3. Timely measurement of the respective quality attribute.

In order to determine whether at-line RPLC met the criteria of an in-process monitoring tool, DTT concentration and the solubilization time of HRP IBs were varied in a DoE approach (Table 2). DTT was required during solubilization in order to keep the cysteines (eight contained in HRP) in a reduced state and therefore prevent aggregation caused by an intermolecular disulfide bridge formation. It was expected that DTT concentration and the solubilization time would influence the titer of monomeric HRP in the solubilizate.

The key quality attribute (=DoE response) was defined as the concentration of monomeric HRP. We hypothesized that the enzymatic activity after refolding would directly correlate with the concentration of monomeric HRP in the solubilizate. The aggregated HRP was believed to result in a structure not applicable for refolding whereas monomeric HRP was defined as completely reduced and denatured during solubilization. Therefore, a monomeric HRP titer was chosen as the target response for the DoE. To test whether both RPLC and SDS-PAGE could predict the targeted solubilization for tailored refolding, all solubilizates were refolded and the volumetric activity (U/mL) after refolding was recorded.

**Figure 1:**
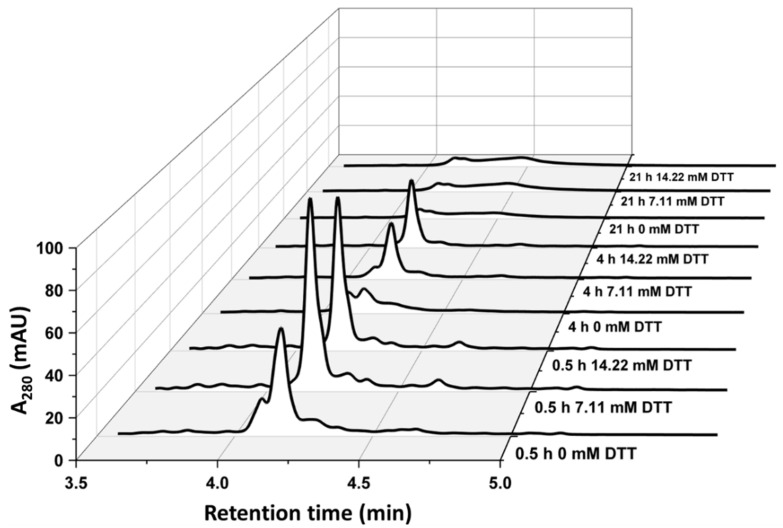
RPLC chromatograms at 280 nm quantifying monomeric HRP eluting at 4.18 min. The results demonstrate the trends of solubilization at three applied DTT concentrations (0 mM, 7.11 mM and 14.22 mM) for 0.5 h of solubilization, 4 h of solubilization and 21 h of solubilization. After 21 h of solubilization, a strong degradation of the target peak in the solubilizate is visible.

Figure 1 shows that the solubilizate containing no DTT displayed an additional peak before the HRP target peak (at a 4.1 min retention time). This peak could already be monitored after 0.5 h of solubilization and increased with longer solubilization times. A complete degradation of the target peak could be observed after 21 h of solubilization independent of the supplied DTT concentration. The pellets resulting from the centrifugation prior to the RPLC analysis after 21 h solubilization increased compared with pellets received from shorter solubilization times, indicating an enhanced protein aggregate formation [7].

**Figure 2:**
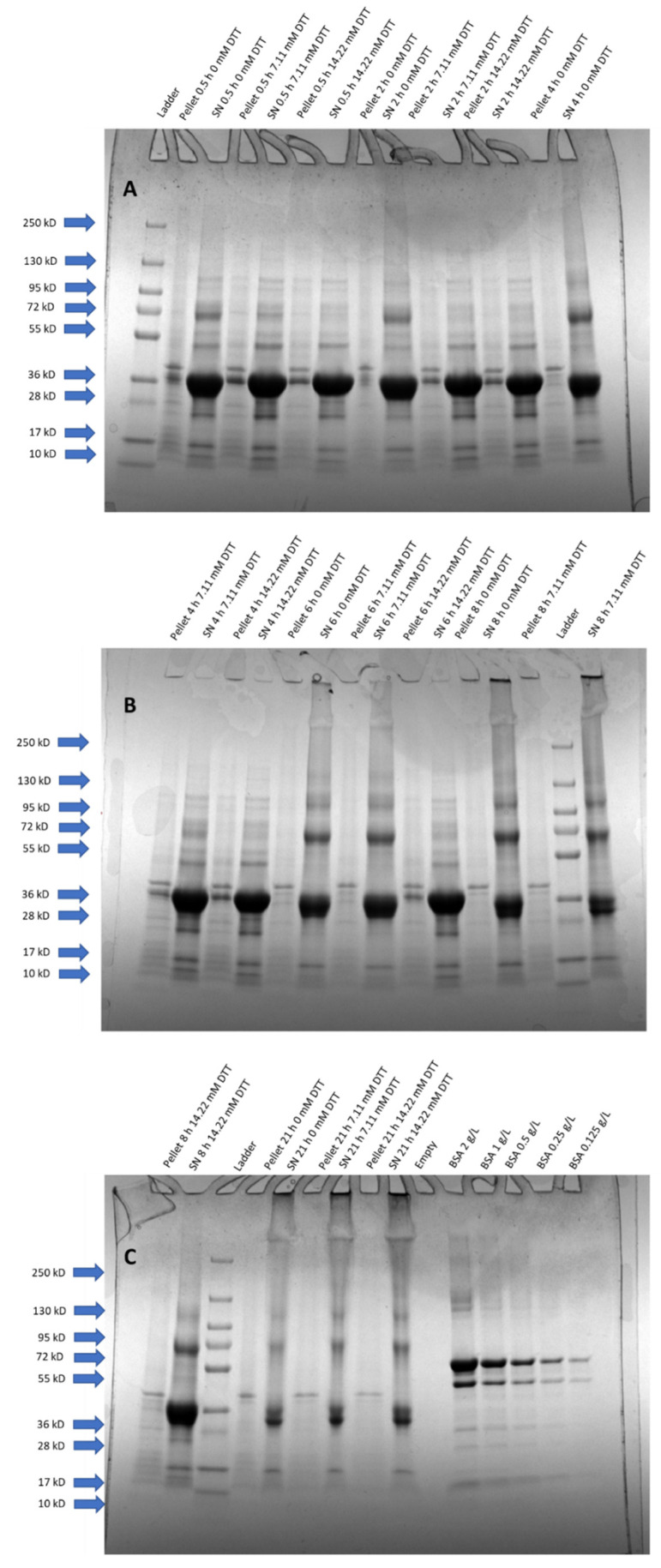
SDS-PAGE analysis of HRP solubilizates with varying DTT concentrations and time factors as depicted in Table 2; non-glycosylated HRP is visible at 34 kDa. The potential dimer formed due to the intermolecular disulfide bridge formation can be seen at 68 kDa. The samples are displayed in the following order from (**A**–**C**): pellet and supernatant (SN) for each time-point varying the three altered DTT concentrations. Additionally to samples, in (**C**), a standard protein calibration with BSA was performed. Protein ladders were added to confirm protein size.

For the SDS-PAGE analysis, the HRP target band was found at 34 kDa (Figure 2). We hypothesized that the protein band at approximately 68 kDa was a product dimer due to an intermolecular disulfide bridge formation. The 68 kDa band tended to increase with extended solubilization times, especially beyond 4 h and for experiments without DTT. In good agreement with the RPLC measurements, we found that HRP was substantially degraded after 21 h of solubilization (Figure 2C). It was therefore concluded that both factors varied for this DoE (DTT concentration and solubilization time) had an influence on the quality attribute “monomeric HRP concentration” in the solubilizate. Furthermore, SDS-PAGE and RPLC were able to measure these changes, with RPLC being applicable as an at-line monitoring tool due to its short analysis time of less than 10 min.

**Figure 3:**
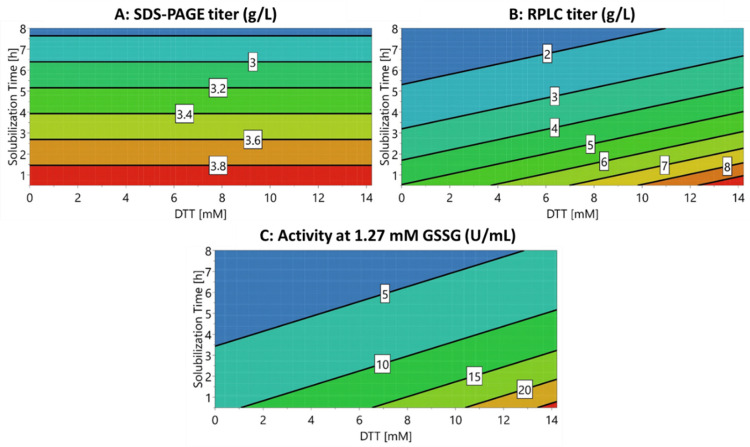
MODDE contour plots with the two factors of DTT concentration on the X-axis and the solubilization time on the Y-axis. The following responses are shown: (**A**) Monomeric HRP concentration in the solubilizate (g/L) analyzed using SDS-PAGE. (**B**) Monomeric HRP concentration in the solubilizate (g/L) analyzed using RPLC. (**C**) Effect of the different solubilization conditions on the volumetric activity (U/mL) after refolding, which was performed at constant GSSG conditions of 1.27 mM.

A multivariate data approach was applied in order to quantify the effects of the varied DTT concentrations and solubilization times on the monomeric HRP concentration during solubilization. The contour plots (i.e., model responses) of the multivariate data analysis for SDS-PAGE and RPLC in solubilization and the enzymatic activity after refolding are shown in Figure 3. All concentrations of HRP during solubilization (including respective purity) for the shown experiment are listed in Supplementary Table S1 for both SDS-PAGE and RPLC. The results for 21 h of solubilization (Figure 1 and Figure 2, Supplementary Table S1) were excluded from the model as they led to a response distortion due to product degradation. The model terms (R^2^ and Q^2^) are summarized in Supplementary Figure S1. Furthermore, ANOVA plots for the responses and significant factors used for the models are shown in Supplementary Figures S2 and S3, respectively.

The results obtained for the model responses varied between the SDS-PAGE and the RPLC analysis for the conducted DoE (Figure 3A,B). Surprisingly, the SDS-PAGE analysis indicated that the monomeric HRP concentration was solely dependent on the solubilization time (Figure 3A). The raw data of both SDS-PAGE and RPLC (Figure 1 and Figure 2) showed that short solubilization times below 4 h were superior over longer solubilization times. However, the raw data also indicated that high DTT concentrations were beneficial for the concentration of monomeric HRP during solubilization whereas lower DTT concentrations led to a lower solubilization yield. This effect was especially true for longer solubilization times. The model prediction for the SDS-PAGE analysis was very low in comparison with the RPLC analysis (Supplementary Figure S1). We hypothesized that this trend occurred due to different separation principles leading to non-linear model responses. The model response for the RPLC analysis with the monomeric HRP concentration indicated that both DTT concentration and the solubilization time had a significant influence (Figure 3B).

The effects on the product purity during solubilization can be found in Supplementary Table S1 and Figure S4 for both SDS-PAGE and RPLC, indicating the same trends as for the monomeric HRP concentration shown in Figure 3A,B. The differences in the total titer between the SDS-PAGE analysis and RPLC might result from the high sample concentrations chosen for SDS-PAGE. This was done to determine the impurities and monomeric HRP titer in the SDS-PAGE analysis. For the RPLC analysis, monomeric HRP and purity could be well assessed within one chromatogram.

To assess which analytical method was better suited for refolding yield prediction, the volumetric activity (U/mL) after refolding was determined (Figure 3C). The GSSG concentration was varied according to previous experiments [37]; however, no alterations in the model trend were obtained (Supplementary Table S1). The effects are exemplarily shown for 1.27 mM GSSG in Figure 3C. The enzyme activity after refolding was found to be highest at short solubilization durations (0.5 h, Figure 3C) and an increase in DTT concentration led to increased enzyme activity. Hence, the enzyme activity showed the same trends as RPLC solubilizate prediction (Figure 3B,C) while solubilizates quantified via SDS-PAGE led to a different prediction than enzymatic activity after refolding (Figure 3A,C).

Therefore, the presented RPLC method met the criteria required for an at-line monitoring tool for the solubilization of HRP IBs. In comparison with the commonly used SDS-PAGE, the models based on the RPLC data were able to predict the influence of DTT concentration and time during solubilization on the subsequent refolding step and the refolding yield correctly. Furthermore, due to the versatility and short analysis time [18], RPLC could be applied as a suitable technique for the in-process control of IB processes.

### 3.2. Demonstration of At-Line RPLC for IPC

The refolding yield highly depends on the protein concentration during refolding with lower protein concentrations favoring higher refolding yields [6,9]. The protein concentration that leads to a maximum space-time yield of a correctly folded protein is empirically determined during process development of refolding [37]. The protein concentration in solubilization is commonly controlled by a fixed amount of wet IB weight dissolved in a solubilization buffer and a subsequent fixed dilution in the refolding buffer [10]. However, this approach is highly dependent on a rigid HRP titer per wet IB weight as well as a constant solubilization yield. It guarantees the desired concentration of the target product during solubilization and the subsequent refolding. In-process monitoring of the target protein concentration in the solubilizate is necessary to counteract batch to batch variations derived from upstream processing (USP) and varying yields during solubilization.

In order to evaluate if the presented RPLC method was suited for the IPC, we conducted solubilization and refolding experiments comparing two IB batches. Early DSP until the refolding step were kept constant for both IB batches. The refolding was performed by two different approaches:

1. Solubilizates were diluted at a fixed ratio of 1:40, as previously determined [37];
2. Solubilizates were diluted according to the product quantity assessed by RPLC (Table 3).

As shown in Table 3, the concentration of monomeric HRP in the solubilizate varied from Batch 1 (5.27 g/L) to Batch 2 (2.35 g/L). This led to an over 50% decrease of enzymatic activity after refolding for Batch 2 if the empirically determined fixed dilution of 1:40 was applied. When using the RPLC method as an in-process control tool, the dilution for Batch 2 could be adapted to 1:17. While the refolding yield for Batch 2 only decreased minimally when adjusting the dilution from 1:40 to 1:17 (Table 3), the refolding buffer volume could be reduced by more than 50%. Therefore, using a correction via RPLC, the 54% variation caused by USP could be reduced to only 11%. However, for the IPC in solubilization and refolding, the following factors still needed to be considered to elucidate the activity deviation of 11%:

1. The corrected dilution in refolding led to a variation in the redox system because a higher DTT carry-over occurred at lower dilutions. This shift of the redox system potentially influences the refolding yield [37].
2. For the demonstration purpose of the IPC via RPLC, drastic deviations from the USP were targeted (i.e. 54% of titer deviation). However, the protein concentration adjustment in refolding via at-line RPLC from solubilization might be even less error-prone for smaller deviations.
3. Furthermore, standard deviations resulting from the RPLC measurements and enzymatic assay could explain further deviations (Table 3).

In the case of HRP IBs, the presented RPLC method was applied successfully as an IPC tool. Based on the rapid analysis time of the developed RPLC method, the deviations caused by the USP could be monitored at-line and the dilution was adapted to minimize the deviations during the refolding step. In addition, the received IB fingerprint (i.e., impurity monitoring) obtained via RPLC could provide valuable information using a reference impurity pattern for industrial applications.

## 4. Conclusions

The solubilization of IBs prior to refolding is essential to obtain the desired protein conformation and protein concentrations in refolding. In this study, we developed an at-line RPLC method to monitor the target protein concentration during the solubilization unit operation of IB processing. DTT concentration and the solubilization time were varied for HRP IBs and the monomeric HRP concentration was recorded using SDS-PAGE and RPLC. The short analysis time (8.1 min), facile sample preparation and the high accuracy of RPLC (as demonstrated for the generic method [18]) allowed for a precise prediction of the monomeric HRP concentration on the refolding yield. The results thus favored the RPLC analysis over the SDS-PAGE analysis as the former could also be used for in-process monitoring.

Moreover, we demonstrated the IPC making use of the developed RPLC method, determining USP alterations. As downstream operations are performed sequentially in industry, subsequent unit operations are influenced by initial deviations. In this study, the protein concentration in refolding could be adapted by adjusting the dilution factor based on the at-line RPLC analysis. This allowed for a more robust refolding process against the deviations contrived from the USP and a reduction of the refolding buffer compared with empirical dilutions thus facilitating a more economic process.

Concluding, the developed RPLC method can be applied to accelerate process development in IB solubilization and for in-process monitoring therefore allowing IPC, which facilitates tailored IB refolding.

## Figures and Tables

**Table 1:** Gradient used for the RPLC analysis, with eluent A being ultrapure water and eluent B acetonitrile both supplemented with 0.1% (v/v) trifluoroacetic acid.

Time (min)	Percent Eluent B (%)
0	25
3.1	62
5.1	62
5.2	25
8.1	25

**Table 2:** Full factorial experimental design for HRP solubilization. The listed DTT concentrations were used in combination with each listed time-point.

Time (h)	DTT Concentrations (mM)
0.5	0
2	7.11
4	14.22
6	-
8	-
21	-

**Table 3:** HRP concentration in the solubilizate was determined via RPLC. Furthermore, the dilution in the refolding buffer as well as the activity after refolding are given for the two different IB batches. Two different dilutions were done for Batch 2: (1) solubilizate was diluted with a fixed dilution (1:40) and (2) dilution was adapted to achieve the same HRP concentration as for Batch 1 (1:17).

IB Batch	c(HRP) (g/L) in Solubilization	Applied Dilution	Activity (U/mL) after Refolding
Batch 1, Fixed Dilution	5.27 ± 0.11	1:40	89.7 ± 6.0
(1) Batch 2, Fixed Dilution	2.35 ± 0.05	1:40	41.9 ± 2.8
(2) Batch 2, IPC via RPLC	2.35 ± 0.05	1:17	79.8 ± 5.4

## Data Availability

The data presented in this study are available on request from the corresponding author. The data are not publicly available due to cooperation with an industrial partner.

## Supplementary Materials

The following are available online at https://www.mdpi.com/article/10.3390/bioengineering8060078/s1, Table S1: Conditions and raw data for the HRP solubilization DoE. Figure S1: Showing the measure of fit (R^2^) of the model and the model predictability (Q^2^) for the multivariate data analysis (based on multiple linear regression) conducted. SDSHRP conducts for titer measurements in solubilization performed with SDS-PAGE whereas SDSpurity shows the model for SDS impurity measurement in solubilization. It can be seen that these models show a low model predictability and a low measure of fit compared to other models in supplementary Figure S2. RPHRP and RPpurity copes for solubilization models of HRP titer and impurities respectively. Models after refolding are abbreviated according to their GSSG concentration i.e., 0 mM GSSG in refolding = vAct 0 mM GSSG, 1.27 mM GSSG in refolding = vAct 1.27 mM GSSG and 2.54 mM GSSG in refolding = vAct 2.54 mM GSSG; All model except for SDS-PAGE prediction (i.e. SDSHRP and SDSImp) show a R2 close to 0.8 and Q2 close to 0.7 and can thus be regarded as models describing input data appropriately. Figure S2: ANOVA plots are displayed for utilized responses. SDSHRP displays the concentration of monomeric HRP measured using SDS-PAGE, whereas SDSpurity shows the purity of the monomeric HRP analyzed via SDS-PAGE. RPHRP displays the concentration of monomeric HRP measured using RPLC and RPpurity shows the purity of monomeric HRP measured using RPLC analysis. vAct 1.27 mM GSSG shows the volumetric activity [U/mL] after refolding with 1.27 mM GSSG contained in the refolding buffer. For each response, SD-regression shows the variation of the response explained by the model while the RSD shows the variation of the response which is not explained by the model. Both values are adjusted for the respective degrees of freedom. RSD*sqrt(F(crit)) shows RDS multiplied by the square root of the critical F (statistically significant at the 95% confidence level). Figure S3: Significant factors contributing for the models for the used responses. SDSHRP displays the concentration of monomeric HRP measured using SDS-PAGE, whereas SDSpurity shows the purity of the monomeric HRP analyzed via SDS-PAGE. RPHRP displays the concentration of monomeric HRP measured using RPLC and RPpurity shows the purity of monomeric HRP measured using RPLC analysis. vAct 1.27 mM GSSG shows the volumetric activity [U/mL] after refolding with 1.27 mM GSSG contained in the refolding buffer. Abbreviated factors are: Tim is the solubilization time [h], DTT is the DTT concentration during solubilization [mM]. For both SDS-PAGE responses (SDSHRP and SDSpurity), only the solubilization time was identified as a significant factor. For the RPLC responses as well as the volumetric activity after refolding both the DTT concentration during solubilization and the solubilization time were significant factors. Figure S4: Comparison of the two quality attributes monomeric HRP concentration [g/L] and purity [%] measured with SDS-PAGE and RPLC, respectively. MODDE contour plots with the two factors DTT concentration on the X-axis and the solubilization time on the Y-axis. The following responses are shown: A: Monomeric HRP concentration in the solubilizate [g/L] analyzed using SDS-PAGE. B: Purity [%] of the monomeric HRP concentration in the solubilizate analyzed via SDS-PAGE. C: Monomeric HRP concentration in the solubilizate [g/L] analyzed using RPLC. D: Purity [%] of the monomeric HRP concentration in the solubilizate analyzed via RPLC.
