# Supplementary material for: At-Line Reversed Phase Liquid Chromatography for In-Process Monitoring of Inclusion Body Solubilization
Source: Bioengineering (Basel). 2021 Jun 7;8(6):78. doi: 10.3390/bioengineering8060078 (PMC8228044; doi:10.3390/bioengineering8060078)
Supplement: Supplementary file 1 [file bioengineering-08-00078-s001.zip › bioengineering-1233133-supplementary.pdf]

bioengineering

MDPI

At-line reversed phase liquid chromatography for in-process monitoring of inclusion body solubilization

Julian Ebner [1], Diana Humer [1], Robert Klausser [1], Viktor Rubus [1], Reinhard Pell [2], Oliver Spadiut [1] Julian Kopp [1,*]

[1] Research Division Integrated Bioprocess Development, Institute of Chemical, Environmental and Bioscience Engineering, Vienna University of Technology, 1060 Vienna, Austria; julian.ebner@tuwien.ac.at (J.E.); diana.humer@tuwien.ac.at (D.H.); robert.klausser@tuwien.ac.at (R.K.); viktor.rubus@tuwien.ac.at (V.R.); oliver.spadiut@tuwien.ac.at (O.S.)

[2] SANDOZ GmbH, Mondseestrasse 11, 4866 Unterach, Austria; reinhard.pell@gmx.at

* Correspondence: julian.kopp@tuwien.ac.at

Supplementary Table S1:

Conditions and raw data for the HRP solubilization DoE. Product purity was assessed as area- percentage for RPLC and SDS-PAGE using equation 1:

$$\text{EQ1: } Solubilizate\ purity\ [\%] = \frac{Target\quad protein_{Area}[-]}{Target\ protein_{Area}[-] + Impurities_{Area}[-]}$$

The volumetric activities were measured after refolding the listed solubilization conditions with a concentration of 1.27 mM GSSG. Measurements were performed in triplicates and standard deviation was <12% in all cases.

DTT [mM]	Solubilization time [h]	HRP (SDS-PAGE) [g/L]	Purity HRP (SDS-PAGE) [%]	HRP (RP-HPLC) [g/L]	Purity HRP (RP-HPLC) [%]	Volumetric activity [U/mL]
0	0.5	3.97	53.2	4.98	41.4	5.0
7.11	0.5	3.28	50.4	8.04	51.8	19.1
14.22	0.5	4.18	58.0	6.30	56.8	17.0
0	2	4.16	59.6	3.90	36.1	6.6
7.11	2	3.77	52.3	6.12	55.1	17.5
14.22	2	3.52	43.3	6.19	53.7	21.3
0	4	3.23	48.1	2.29	18.0	5.6
7.11	4	3.35	36.4	5.56	41.1	11.0
14.22	4	3.48	39.2	6.24	45.1	16.3
7.11	6	3.20	50.3	1.64	11.5	3.8
14.22	6	3.81	32.9	5.86	45.0	11.5
0	8	2.54	37.3	1.62	12.4	2.8
7.11	8	1.88	29.8	0.80	6.8	1.6
14.22	8	3.14	42.3	2.52	18.9	4.4
0	21	0.87	28.5	0.59	7.1	1.1
7.11	21	0.89	19.6	0.67	6.3	0.6
14.22	21	1.12	19.5	0.68	7.6	0.6

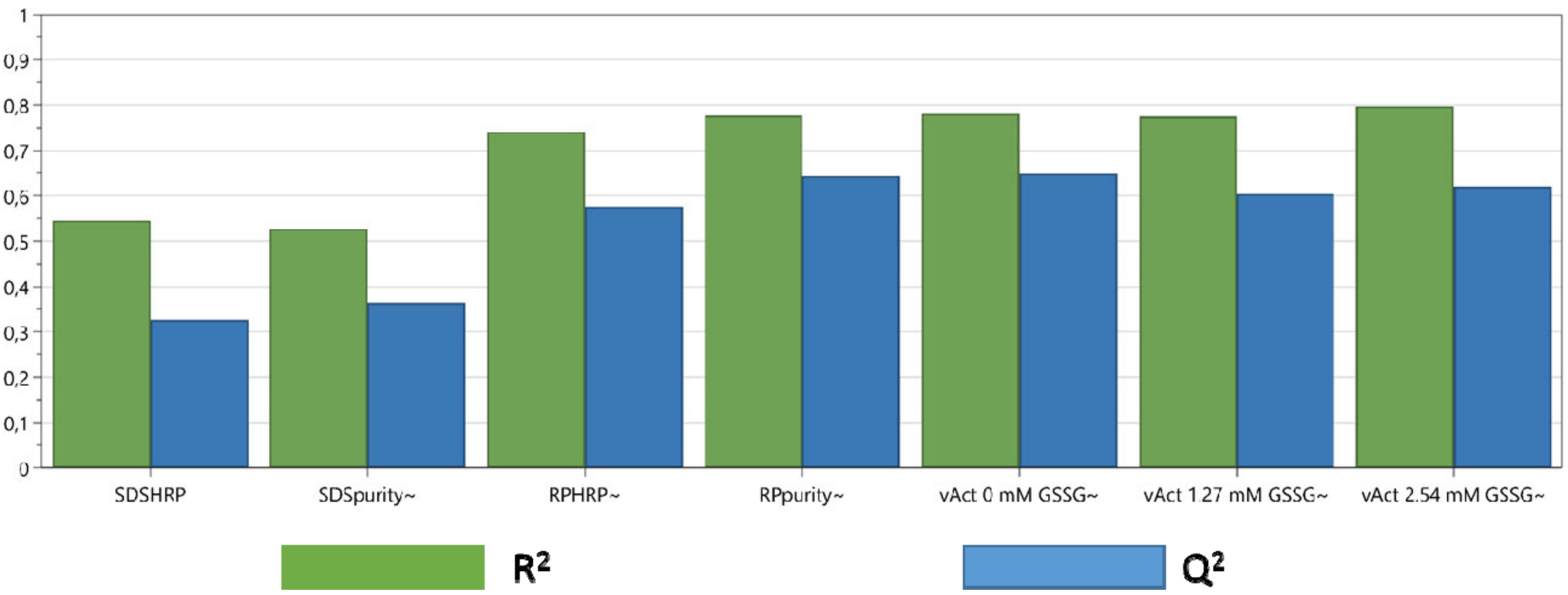

Supplementary Figure S1. Showing the measure of fit (R^2) of the model and the model predictability (Q^2) for the multivariate data analysis (based on multiple linear regression) conducted. SDSHRP conducts for titer measurements in solubilization performed with SDS-PAGE whereas SDSpurity shows the model for SDS impurity measurement in solubilization. It can be seen that these models show a low model predictability and a low measure of fit compared to other models in supplementary figure 2. RPHRP and RPpurity copes for solubilization models of HRP titer and impurities respectively. Models after refolding are abbreviated according to their GSSG concentration i.e. 0mM GSSG in refolding = vAct 0 mM GSSG, 1.27 mM GSSG in refolding = vAct 1.27 mM GSSG and 2.54 mM GSSG in refolding = vAct 2.54 mM GSSG; All model except for SDS-PAGE prediction (i.e. SDSHRP and SDSImp) show a R^2 close to 0.8 and Q^2 close to 0.7 and can thus beregarded as models describing input data appropriately.

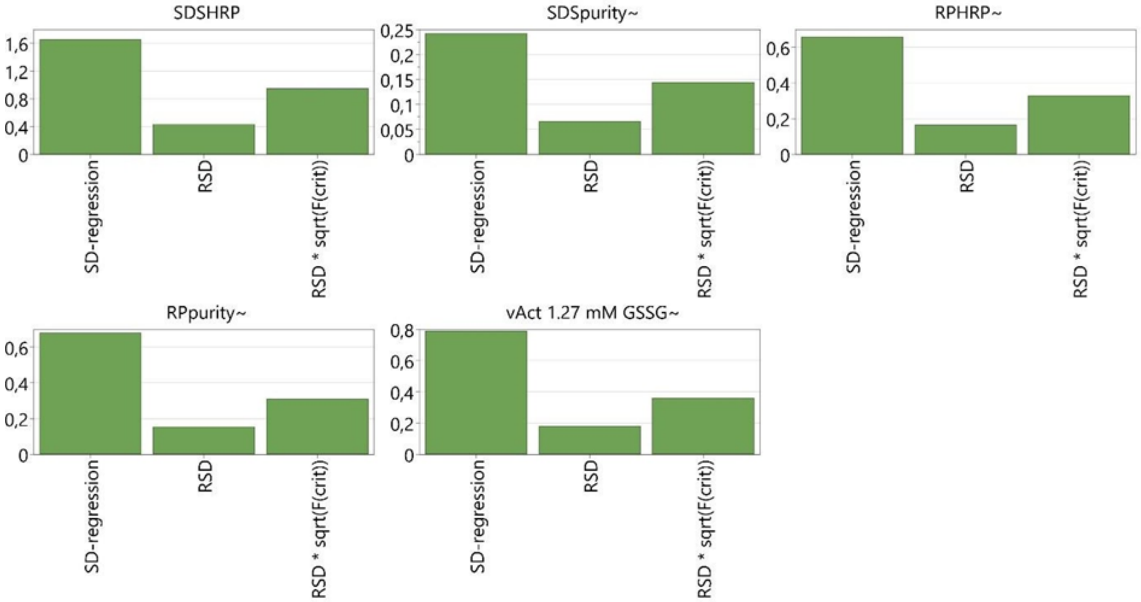

Supplementary Figure S2. ANOVA plots are displayed for utilized responses. SDSHRP displays the concentration of monomeric HRP measured using SDS-PAGE, whereas SDSpurity shows the purity of the monomeric HRP analyzed via SDS-PAGE. RPHRP displays the concentration of monomeric HRP measured using RPLC and RPpurity shows the purity of monomeric HRP measured using RPLC analysis. vAct 1.27 mM GSSG shows the volumetric activity [U/mL] after refolding with 1.27 mM GSSG contained in the refolding buffer. For each response, SD-regression shows the variation of the response explained by the model while the RSD shows the variation of the response which is not explained by the model. Both values are adjusted for the respective degrees of freedom.

RSD*sqrt(F(crit)) shows RDS multiplied by the square root of the critical F (statistically significant at the 95% confidence level).

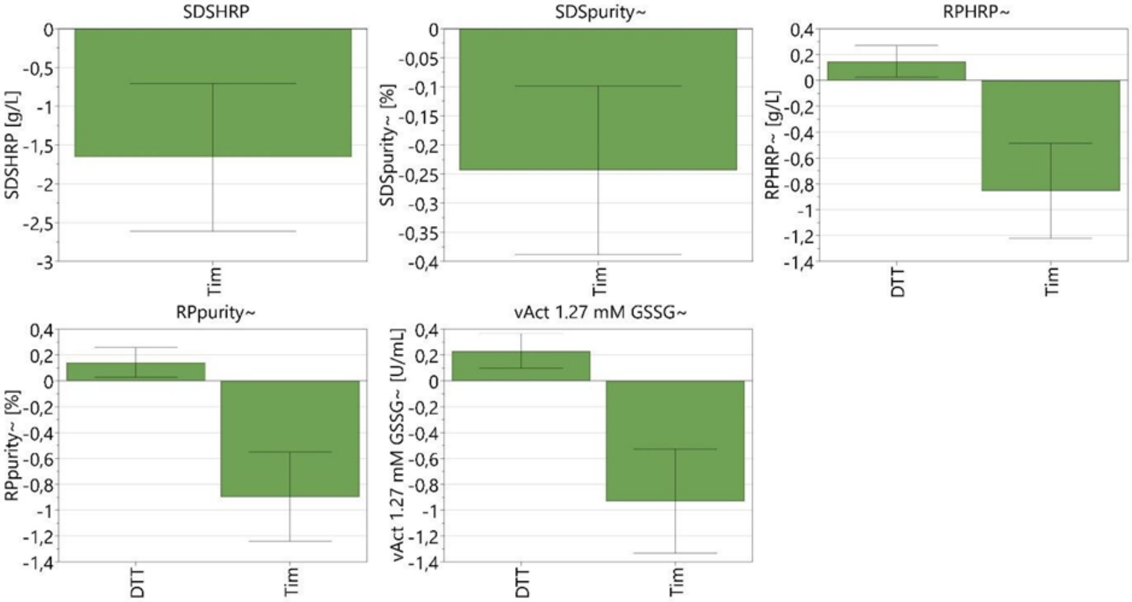

Supplementary Figure S3. Significant factors contributing for the models for the used responses. SDSHRP displays the concentration of monomeric HRP measured using SDS-PAGE, whereas SDSpurity shows the purity of the monomeric HRP analyzed via SDS-PAGE. RPHRP displays the concentration of monomeric HRP measured using RPLC and RPpurity shows the purity of monomeric HRP measured using RPLC analysis. vAct 1.27 mM GSSG shows the volumetric activity [U/mL] after refolding with

1.27 mM GSSG contained in the refolding buffer. Abbreviated factors are: Tim is the solubilization time [h], DTT is the DTT concentration during solubilization [mM]. For both SDS-PAGE responses (SDSHRP and SDSpurity), only the solu-

bilization time was identified as a significant factor. For the RPLC responses as well as the volumetric activity after refolding both the DTT concentration during solubilization and the solubilization time were significant factors.

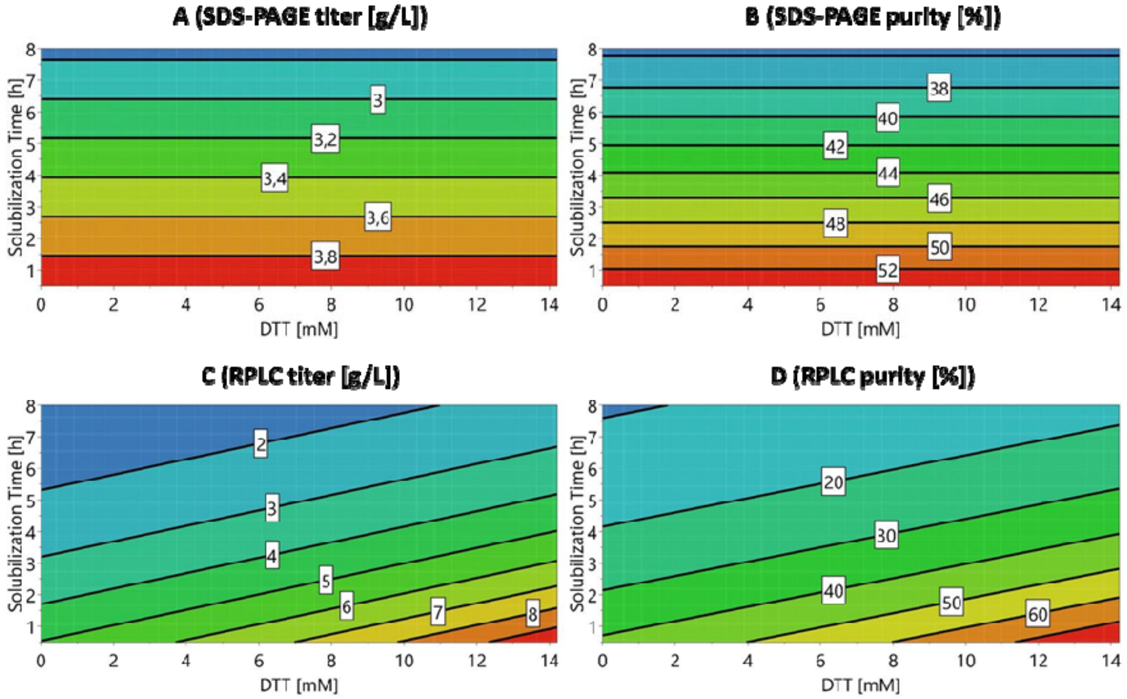

Supplementary Figure S4. Comparison of the two quality attributes monomeric HRP concentration [g/L] and purity [%] measured with SDS-PAGE and RPLC, respectively. MODDE contour plots with the two factors DTT concentration on the X-axis and the solubilization time on the Y-axis. The following responses are shown: A: Monomeric HRP concentration in the solubilizate [g/L] analyzed using SDS- PAGE. B: Purity [%] of the monomeric HRP concentration in the solubilizate analyzed via SDS-PAGE. C: Monomeric HRP concentration in the solubilizate [g/L] analyzed using RPLC. D: Purity [%] of the monomeric HRP concentration in the solubilizate analyzed via RPLC.
